# Simultaneous Calibration of Odometry and Head-Eye Parameters for Mobile Robots with a Pan-Tilt Camera

**DOI:** 10.3390/s19163623

**Published:** 2019-08-20

**Authors:** Nachaya Chindakham, Young-Yong Kim, Alongkorn Pirayawaraporn, Mun-Ho Jeong

**Affiliations:** 1 Department of Control and Instrumentation Engineering; Kwangwoon University, Kwangwoon-ro 1-gil 60, Nowon-gu, Seoul 01890, Korea; 2 Research and Development Department; Thinkware Visual Technology, 240 Pangyoyeok-ro, Bundang-gu, Gyeonggi-do, Seongnam-si 463-400, Korea

**Keywords:** mobile robot kinematics, odometry calibration, head-eye calibration, simultaneous mobile robot calibration

## Abstract

In the field of robot navigation, the odometric parameters, such as wheel radii and wheelbase length, and the relative pose of the optical sensing camera with respect to the robot are very important criteria for accurate operation. Hence, these parameters are necessary to be estimated for more precise operation. However, the odometric and head-eye parameters are typically estimated separately, which is an inconvenience and requires longer calibration time. Even though several researchers have proposed simultaneous calibration methods that obtain both odometric and head-eye parameters simultaneously to reduce the calibration time, they are only applicable to a mobile robot with a fixed camera mounted, not for mobile robots equipped with a pan-tilt motorized camera systems, which is a very common configuration and widely used for wide view. Previous approaches could not provide the *z*-axis translation parameter between head-eye coordinate systems on mobile robots equipped with a pan-tilt camera. In this paper, we present a full simultaneous mobile robot calibration of head–eye and odometric parameters, which is appropriate for a mobile robot equipped with a camera mounted on the pan-tilt motorized device. After a set of visual features obtained from a chessboard or natural scene and the odometry measurements are synchronized and received, both odometric and head-eye parameters are iteratively adjusted until convergence prior to using a nonlinear optimization method for more accuracy.

## 1. Introduction

Robot navigation is one of the key challenges facing the field of mobile robotics. This is because mobile robots are required to drive themselves through a given environment using the information gathered from their sensors. These sensors include proprioceptive sensors such as motor-speed sensors, wheel-load sensors, joint-angle sensors, battery-voltage sensors and the inertial measurement unit (IMU), which produces a type of data called odometric data. Additionally, these robots are fitted with exteroceptive sensors such as image-feature sensors, distance sensors, light-intensity sensors, sound-amplitude sensors, and global positioning system (GPS) sensors. However, robot localization is typically inaccurate due to the uncertainty associated with measurement errors during robot configuration. Although the robot configuration data, such as wheel radii and wheelbase length, can be obtained simply from the robot specifications or by manual measurement, the actual parameters can be dissimilar in practice. This is due to systematic errors such as manufacturing errors, assembly errors, tire pressure variations, and load variations that reduce the precision associated with the movement of the mobile robot. It is therefore necessary to estimate these odometric parameters to improve the robot’s operational precision.

Antonelli’s calibration method [1] uses the least-squares technique to observe a linear mapping between the unknowns and the measurements. It aims to identify a 4-parameter model, while the modified version [2] estimates the physical odometric parameters and yields a 3-parameter model without any requirement for a predefined path. Some researchers, such as [3,4], have reduced the cumulative error in odometry by considering the coupled effect of errors in diameter, wheelbase errors, and scaling errors using a popular odometry calibration method for wheeled mobile robots, developed at and named the University of Michigan Benchmark test (UMBmark) [5]. Some trials required a static wireless sensor network and GPS devices to be equipped on mobile robots to correct for the odometry errors [6,7]. However, this approach is disadvantageous in terms of cost-effectiveness.

Nowadays, mobile robots equipped with cameras which provide single color images, stereo images, or depth images are widely used in the fields of robot navigation, reconstruction, and mapping. Using this information, the robot can perform more precise and varied tasks. However, the relationship between the camera and robot in terms of 3D position and orientation is also important for accurate operation. Shiu and Ahmad [8] introduced a solution for rigid transformation between the sensor and the robot, calculated in the form AX = XB. Hand-eye calibration, as proposed by Tsai and Lenz [9], is a similar, albeit more efficient solution which does not depend on the number of images. In mobile robots, the 3D position and orientation of the camera relative to the robot’s base is considered instead. Kim et al. [10] found that the head-eye transformation between the robot’s coordinate system and the camera’s coordinate system can be estimated simply and accurately by using the minimum variance technique, which is resistant to noisy environments.

In practical applications, the odometric parameters, such as wheel radii and wheelbase length, and the head-eye parameters are typically estimated separately, which requires a longer calibration time and increased inconvenience due to redundancy in these methods. To avoid the disadvantages, Antonelli et al. [11] have proposed a simultaneous calibration method that performs both odometry and the head-eye calibrations simultaneously. Since the method required only the synchronized measurement of odometric data and visual features, it was successful in terms of reducing the calibration time and improving efficiency in the mobile robot calibration. However, their approach is only applicable to the mobile robot on which a fixed camera is mounted, while recent mobile robots are equipped with pan-tilt motorized camera systems for wide view. This caused incomplete estimation of the head-eye parameters, that is, the method could not provide the *z*-axis translation parameter. Shusheng Bi et al. [12] presented an improved version of [11] in terms of accuracy, but still did not overcome the problem. Hengbo Tang et al. [13] solved the problem by taking the advantage of the planar constraints of the landmarks. Despite their accurate estimation of the head-eye parameters and the odometric parameters, there is a clear limitation in the sense that a very constrained environment is needed and several recognizable landmarks must be premeasured and fixed.

In this paper, we present a full mobile robot calibration of head-eye and odometric parameters, building on [14]. The full six parameters (rotation and translation) in the head-eye calibration and the three parameters (wheel radii and wheelbase length) in the odometry calibration are simultaneously estimated. The mobile robot equipped with a mono or stereo camera moves while the camera mounted on the pan-tilt motorized device is capturing chessboards or natural scenes. After simply planned robot movements, the full mobile robot calibration algorithm is performed using both odometry measurements and visual features such as chessboard’s corners or natural feature points from a stereo camera, which are obtained by Speeded-Up Robust Feature (SURF) [15]. The head-eye and odometric parameters are iteratively adjusted to obtain the values, which are searched as a good starting point close to the ground truth please confirm intended meaning is retained. and then finally fine-tuned with the direct search-based optimization as Powell’s method [16]. The remainder of this paper is organized as follows: Section 2 describes our mobile robot configuration and the relationship between each joint. Section 3 proposes an iterative-based calibration method for mobile robot having a camera mounted on a motorized neck. Section 4 presents our experimental results, and the conclusion is summarized in Section 5.

## 2. Mobile Robot Configuration

### 2.1. Robot Coordinate System

**Figure 1:**
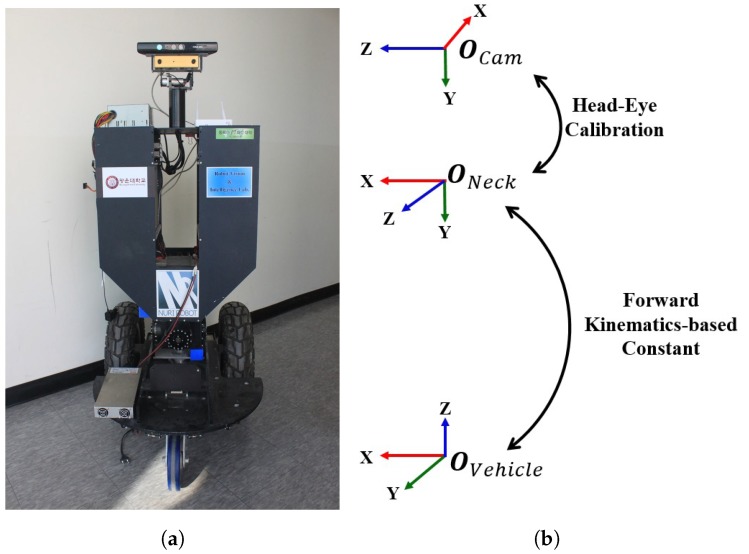
Mobile robot configuration. (**a**) Robot having a pan-tilt neck equipped with a camera (front-view); (**b**) coordinate system of mobile robot configuration (side-view).

The mobile robot configuration in Figure 1a can be understood in terms of an overview of the coordinate system of a mobile robot with a pan-tilt camera, as depicted in Figure 1b. It consists of the vehicle coordinate (robot’s base), $O_{Vehicle}$; the neck coordinate includes a pan-tilt joint, ($O_{Neck}$), with a camera mounted on the top as camera coordinate, $O_{Cam}$. The relation between the vehicle system $O_{Vehicle}$ and $O_{Neck}$ is estimated using Denavit–Hartenberg parameters (DH parameters) depending on the mobile robot configuration. The rotation from the neck to camera and the rotations between robot’s bases are estimated in Section 3.2. The remain parameters are calculated in Section 3.4.

### 2.2. Robot Wheel Parameters

**Figure 2:**
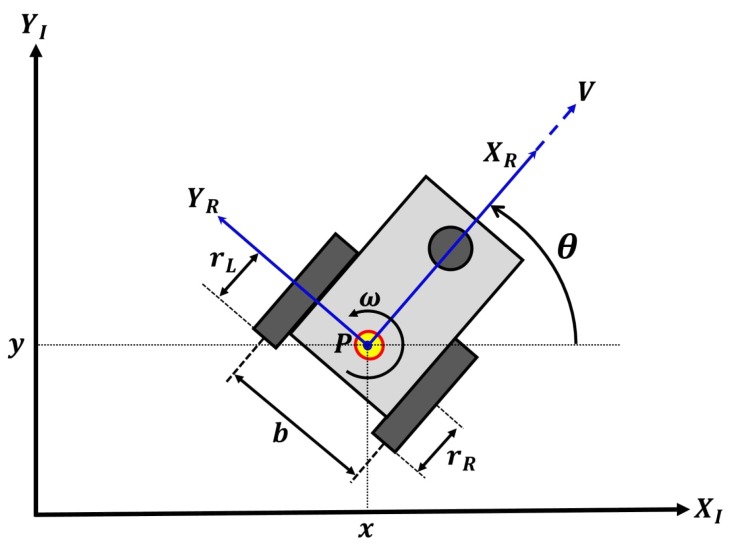
Mobile robot odometry and its relevant variables.

In the field of robot navigation, one of the important parameters for mobile robot calibration is the wheel parameters, which are the radii of the left and right robot wheels and the baseline (the axle length between left and right wheel), as shown in Figure 2. The robot kinematics can be expressed as

(1) $$\begin{array}{cc} \dot{x} & =\upsilon \cos(\theta ),\\ \dot{y} & =\upsilon \sin(\theta ),\\ \dot{\theta } & =\omega , \end{array}$$

where υ, ω, and θ are the velocity, angular velocity, and the orientation of the mobile robot, respectively, as depicted in Figure 2. These parameters can be obtained by using the following equation:

(2) $$\begin{array}{cc} \upsilon & =\frac{r_{R}}{2}\omega_{R}+\frac{r_{L}}{2}\omega_{L},\\ \omega & =\alpha_{R}\omega_{R}+\alpha_{L}\omega_{L}, \end{array}$$

where $\alpha_{R}=\frac{r_{R}}{b}$ and $\alpha_{L}=-\frac{r_{L}}{b}$. The wheel parameters as $r_{R}$, $r_{L}$, and *b* are right and left wheel radii and the length of the baseline, respectively. $\omega_{R}$ and $\omega_{L}$ are angular velocities, which are calculated using the encoder on the right and left wheels. The ratio between the radii of wheels and the length of wheelbase is represented in terms of intermediate parameters, ($\alpha_{R},\alpha_{L}$), which are used in the calibration process instead of the real wheel parameters.

The rotational angle of the mobile robot from frame *i* to frame *j*, by $t_{i}$ = 0 and $t_{j}$ = *t*, can be obtained through the integral of Equation (2) with respect to time as follows:

(3) $$\begin{array}{cc} \theta \left(t\right) & =\alpha_{R}\int_{0}^{t}\omega_{R}\left(\tau \right)d\tau +\alpha_{L}\int_{0}^{t}\omega_{L}\left(\tau \right)d\tau ,\\ & =\alpha_{R}\phi_{R}\left(t\right)+\alpha_{L}\phi_{L}\left(t\right), \end{array}$$

where $\phi_{R}\left(t\right),\phi_{L}\left(t\right)$ are the encoder positions of the right and left wheels, respectively.

## 3. Simultaneous Calibration for Mobile Robot with Pan-Tilt Camera

In this section, we describe the proposed calibration method separately in six parts. The closed-loop transformation of the camera and the robot base to the robot neck between any frames *i* and *j*, where *i* = 1, …, *N* − 1 and *j* = *i* + 1 are concisely depicted in Section 3.1. Since a set of captured images, $I_{n=1}^{n=N}$, and the calibration data set (such as rotating angles of wheels ($\phi_{R_{i}},\phi_{L_{i}}$), and transformation from robot base coordinate to robot’s neck coordinate, $T_{N_{i}}^{V_{i}}$) are obtained once as the calibration input data. Section 3.2, Section 3.3, and Section 3.4 explain how to use these data to obtain the head-eye rotation, intermediate wheel parameters, and head-eye translation, including the actual wheel parameters, respectively. These processes are estimated iteratively until the value of all parameters converge, as described in Section 3.5. Finally, Section 3.6 describes the non-linear optimization method that increases the accuracy of the calibration results.

### 3.1. Closed-Loop Transformations

**Figure 3:**
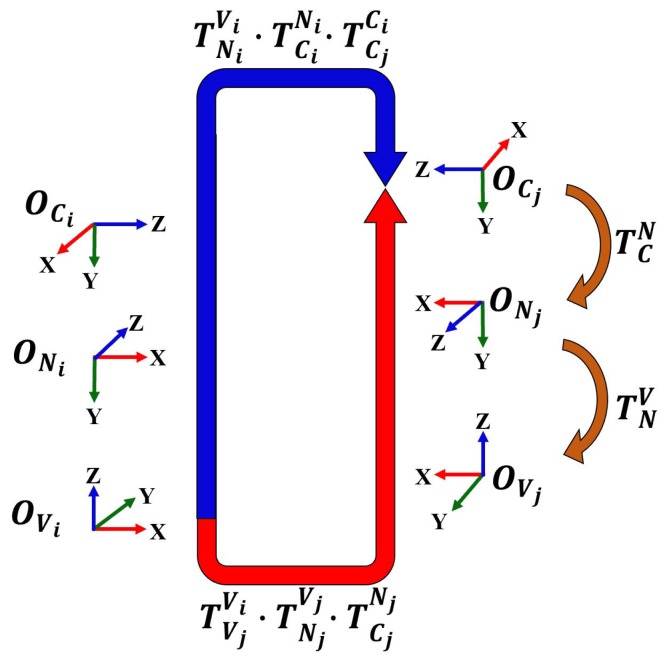
Closed-loop transformation between any frame *i* and *j*.

Let us now consider the homogeneous transformation of different vehicle poses from frame *i* to *j*, as shown in Figure 3. The abbreviations $O_{C},O_{N},O_{V}$ are the camera, neck, and vehicle coordinate systems, respectively. The closed-loop diagram can be represented by Equation (4).

(4) $$T_{N_{i}}^{V_{i}}T_{C_{i}}^{N_{i}}T_{C_{J}}^{C_{i}}=T_{V_{j}}^{V_{i}}T_{N_{j}}^{V_{j}}T_{C_{j}}^{N_{j}},$$

where $T_{N}^{V}$ is the physical relationship between $O_{Neck}$ and $O_{Vehicle}$. The head-eye homogeneous transformation is $T_{C}^{N}$. When the camera is directed at the same target or feature, camera motion is represented as $T_{C_{j}}^{C_{i}}$. The robot’s motion between frame *i* and *j* is presented to $T_{V_{j}}^{V_{i}}$. The homogeneous transformation of Equation (4) can be decomposed into rotational and translational terms as follows:

(5) $$R_{N_{i}}^{V_{i}}R_{C_{i}}^{N_{i}}R_{C_{j}}^{C_{i}}=R_{V_{j}}^{V_{i}}R_{N_{j}}^{V_{j}}R_{C_{j}}^{N_{j}},$$

(6) $$R_{N_{i}}^{V_{i}}R_{C_{i}}^{N_{i}}t_{C_{j}}^{C_{i}}+R_{N_{i}}^{V_{i}}t_{C_{i}}^{N_{i}}+t_{N_{i}}^{V_{i}}=R_{V_{j}}^{V_{i}}R_{N_{j}}^{V_{j}}t_{C_{j}}^{N_{j}}+R_{V_{j}}^{V_{i}}t_{N_{j}}^{V_{j}}+t_{V_{j}}^{V_{i}},$$

where *R* is the 3×3 rotation matrix and *t* is 3×1 translation vector. Equation (5) is used to obtain the head-eye rotation parameters, $R_{C}^{N}$, which consist of 3 degrees of freedom (DOF), $(\gamma_{x},\gamma_{y},\gamma_{z}$). Equation (6) refers to the translation of the system, which is used to estimate the head-eye translation ($t_{C,x}^{N},t_{C,y}^{N},t_{C,z}^{N}$) and the actual size of the wheel parameters ($r_{R},r_{L},b$). These parameters are calculated in Section 3.4.

### 3.2. Head-Eye Rotation Estimation

The six parameters in head-eye calibration consisting of three for rotation and another three for the translation, which are necessary and required to be obtained before an operation. In this section, three parameters of the rotation between the robot’s neck and the camera are calculated precisely. A set of visual measurements and robot movement data were obtained by moving the robot and capturing images synchronously and continuously. The rotation between the camera and the robot’s base was estimated accurately, as by Antonelli et al. [11]. They obtained the rotation parameters between the robot’s base and camera using Equivalent angle-axis representation. However, their approach achieved because their mobile robot had a camera equipped on a fixed neck that did not change the relation between the robot’s base and camera. If the relation between the base and the camera was changed during the calibration data collection, the Equivalent angle-axis method could not be used to solve the problem. During collecting the input calibration data of our mobile robot, both the robot and its neck move that means the rotation between the base and the camera are also changed.

In fact, whenever the robot’s neck moves around the pan-tilt axis, the coordinate of the camera mounted on that neck is also moved significantly. Therefore, the relationship between the camera and the robot’s neck is static, which means the subscript *i* of $R_{N_{i}}^{C_{i}}$ can be omitted as $R_{N}^{C}$ = $R_{N_{1}}^{C_{1}}$ = $R_{N_{2}}^{C_{2}}$ = … = $R_{N_{N}}^{C_{N}}$, where *N* is the total number of input images. Moreover, the mobile robot rotation is performed only on a planar. In other words, the mobile robot rotates around *z*-axis only [11]. Hence, $R_{V_{j}}^{V_{i}}$, in Equation (5) can be replaced with $R_{z}\left(\theta \right)$. Assuming the rotation matrices $R_{C_{j}}^{C_{i}}$, $R_{V_{i}}^{N_{i}}$, and $R_{V_{j}}^{V_{i}}$ are known, which are thoroughly described in Section 4, an Equation (5) can be represented in form of AX = XB, and the rotation $R_{N}^{C}$ can be obtained likewise [8,9] as follows:

(7) $$\begin{array}{cc} R_{C_{j}}^{C_{i}}R_{N}^{C} & =R_{N}^{C}R_{V_{i}}^{N_{i}}R_{z}\left(\theta \right)R_{N_{j}}^{V_{j}}, \end{array}$$

where the matrix *X* is the estimated head-eye rotation, $R_{N}^{C}$. The camera rotations, $R_{C_{j}}^{C_{i}}$, are represented to matrix *A*. The remaining variables of the right side $R_{V_{i}}^{N_{i}}R_{z}\left(\theta \right)R_{N_{j}}^{V_{j}}$ are demonstrated to matrix *B*, which *i* = 1, …, N−1 and *j* = 2, …, *N*.

### 3.3. Intermediate Wheel Parameters Estimation

In this section, the linear relationships of intermediate wheel parameters, ($\alpha_{R},\alpha_{L}$), and rotational angle of the robot movement from the previous to current positions prior to capture any image *i*, $\theta_{j}$, which is obtained with Equation (3) using the period time of the robot movement between the previous and current positions, which is θ(1) = 0 as no loss of generality. The change in the rotational angle of the robot’s base from frames *i* to *j*, on a planar $\theta (t_{j})$ is also re-estimated. According to the robot’s movement on a plane, the change in rotational angle about the *z*-axis of the robot’s base coordinates, which is assumed to be perpendicular to the floor, between a pair of consecutive frames is $R_{z}\left(\theta \right)$. In practical applications, the *z*-axis at the robot’s base coordinates from one consecutive frame to another, ($V_{i}$ and $V_{j}$), may not be parallel because of an error in the estimated $R_{C}^{N}$ and the floor plane of any pair of positions are not parallel. Therefore, the rotational angle about the *z*-axis at the robot’s base coordinates between frames *i* and *j* can be calculated using the Euler angle (ZYX) as follows:

(8) $$\begin{array}{cc} \theta_{j} & =\theta \left(t_{j}\right)=Atan2\left(r_{21},r_{11}\right),\;\;\;\;R_{V_{j}}^{V_{i}}=R_{N_{i}}^{V_{i}}R_{C}^{N}R_{C_{j}}^{C_{i}}R_{N}^{C}R_{V_{j}}^{N_{j}}, \end{array}$$

where $r_{21}$ and $r_{11}$ are generic elements of $R_{V_{j}}^{V_{i}}$. If we consider for *N* images, the representation of Equation (3) can be used to obtain the parameters, ($\alpha_{R}$, $\alpha_{L}$), similar to [11], as follows:

(9) $$\begin{array}{c} \left[\begin{array}{c} \theta_{1}\\ \vdots \\ \theta_{N} \end{array}\right]=\left[\begin{array}{c} \phi_{\theta_{1}}\\ \vdots \\ \phi_{\theta_{N}} \end{array}\right]\left[\begin{array}{c} \alpha_{R}\\ \alpha_{L} \end{array}\right]={\bar{\phi }}_{\theta }\left[\begin{array}{c} \alpha_{R}\\ \alpha_{L} \end{array}\right], \end{array}$$

where $\phi_{\theta_{j}}=\left[\begin{array}{cc} \phi_{R}\left(t_{j}\right) & \phi_{L}\left(t_{j}\right) \end{array}\right]$, are obtained from the rotational angles of both wheels from position *i* to *j*, (*i* = 1, …, *N* − 1 and *j* = 2, …, *N*). ${\bar{\phi }}_{\theta }$ is a matrix with N×2 dimensions. The intermediate wheel parameters, ($\alpha_{R}$, $\alpha_{L}$), can be calculated using the linear least squares method as follows

(10) $$\begin{array}{c} \left[\begin{array}{c} \alpha_{R}\\ \alpha_{L} \end{array}\right]={\left({\bar{\phi }}_{\theta }^{T}{\bar{\phi }}_{\theta }\right)}^{-1}\;{\bar{\phi }}_{\theta }^{T}\cdot \left[\begin{array}{c} \theta_{1}\\ \vdots \\ \theta_{N} \end{array}\right] \end{array}$$

### 3.4. Head-Eye Translation and Wheel Parameters Estimation

The head-eye rotation and intermediate parameters have already been obtained in Section 3.2 and Section 3.3. The remaining parameters that are estimated in this section are $t_{C}^{N}$, the head-eye translation vector, and the actual wheel parameters $r_{R},r_{L},b$. The translational and the rotational components of the robot’s base coordinates can be described using the mobile robot kinematic equations as

(11) $$\begin{array}{cc} x_{j} & =x_{i}+\tau \upsilon_{i}\cos\left(\theta_{i}+\tau \omega_{i}/2\right),\\ y_{j} & =y_{i}+\tau \upsilon_{i}\sin\left(\theta_{i}+\tau \omega_{i}/2\right),\\ \theta_{j} & =\theta_{i}+\tau \omega_{i}, \end{array}$$

where *i* and *j* denote the previous and current frames, respectively, and τ is the period of time between frames. Substituting the intermediate wheel parameters and Equation (2) into Equation (11) yields

(12) $$\begin{array}{cc} x_{j} & =x_{i}+\tau \left(-\frac{\alpha_{R}}{2\alpha_{L}}r_{L}\omega_{R,i}+\frac{r_{L}}{2}\omega_{L,i}\right)\cos\left(\theta_{i}+\tau \omega_{i}/2\right),\\ y_{j} & =y_{i}+\tau \left(-\frac{\alpha_{R}}{2\alpha_{L}}r_{L}\omega_{R,i}+\frac{r_{L}}{2}\omega_{L,i}\right)\sin\left(\theta_{i}+\tau \omega_{i}/2\right). \end{array}$$

In fact, $t_{V_{j}}^{V_{i}}$ is the relative translation in x and y directions, which can be rewritten as

(13) $$\begin{array}{cc} \left[\begin{array}{c} \lambda_{1}\\ \lambda_{2} \end{array}\right] & =\frac{\tau }{2}\left[\begin{array}{c} \left(-\frac{\alpha_{R}}{\alpha_{L}}\omega_{R,i}+\omega_{L,i}\right)\cos\left(\theta_{i}+\tau \omega_{i}/2\right)\\ \left(-\frac{\alpha_{R}}{\alpha_{L}}\omega_{R,i}+\omega_{L,i}\right)\sin\left(\theta_{i}+\tau \omega_{i}/2\right) \end{array}\right]r_{L},\\ t_{V_{j}}^{V_{i}} & ={\left[\begin{array}{c} \lambda_{1}\;\;\lambda_{2}\;\;0 \end{array}\right]}^{T}\cdot r_{L}, \end{array}$$

From Equation (13), the translation between two robot base positions can be substituted into Equation (6) representing the relationship between the coordinates as follows:

(14) $$\begin{array}{c} \left(R_{N_{i}}^{V_{i}}-R_{z}\left(\theta \right)R_{N_{j}}^{V_{j}}\right)t_{C}^{N}-{\left[\begin{array}{c} \lambda_{1}\;\;\lambda_{2}\;\;0 \end{array}\right]}^{T}\cdot r_{L}=-R_{N_{i}}^{V_{i}}R_{C}^{N}t_{C_{j}}^{C_{i}}-t_{N_{i}}^{V_{i}}+R_{z}\left(\theta \right)t_{N_{j}}^{V_{j}}, \end{array}$$

where $R_{N_{i}}^{V_{i}}R_{C}^{N}$ and ($R_{N_{i}}^{V_{i}}-R_{z}\left(\theta \right)R_{N_{j}}^{V_{j}}$) are referred to the matrices *A* and *B*, respectively. Thus, Equation (14) can be simplified as

(15) $$\begin{array}{c} Bt_{C}^{N}-\left[\begin{array}{c} \lambda_{1}\\ \lambda_{2}\\ 0 \end{array}\right]r_{L}=-At_{C_{j}}^{C_{i}}-t_{N_{i}}^{V_{i}}+R_{z}\left(\theta \right)t_{N_{j}}^{V_{j}}, \end{array}$$

(16) $$\begin{array}{c} \left[\begin{array}{ccc} B_{11} & B_{12} & B_{13}\\ B_{12} & B_{22} & B_{23}\\ B_{31} & B_{32} & B_{33} \end{array}\right]\left[\begin{array}{c} t_{C,x}^{N}\\ t_{C,y}^{N}\\ t_{C,z}^{N} \end{array}\right]-\left[\begin{array}{c} \lambda_{1}\\ \lambda_{2}\\ 0 \end{array}\right]r_{L}=-\left[\begin{array}{ccc} A_{11} & A_{12} & A_{13}\\ A_{12} & A_{22} & A_{23}\\ A_{31} & A_{32} & A_{33} \end{array}\right]\left[\begin{array}{c} t_{C_{j,x}}^{C_{i}}\\ t_{C_{j,y}}^{C_{i}}\\ t_{C_{j,z}}^{C_{i}} \end{array}\right]-\left[\begin{array}{c} t_{N,x}^{V}\\ t_{N,y}^{V}\\ t_{N,z}^{V} \end{array}\right]+\left[\begin{array}{ccc} C\theta & -S\theta & 0\\ S\theta & C\theta & 0\\ 0 & 0 & 1 \end{array}\right]\left[\begin{array}{c} t_{N,x}^{V}\\ t_{N,y}^{V}\\ t_{N,z}^{V} \end{array}\right] \end{array}$$

from Equation (16), the third component of the vector ${\left[\begin{array}{c} \lambda_{1}\;\;\lambda_{2}\;\;0 \end{array}\right]}^{T}$ is zero, which also makes the third row zero. It can be derived as follows:

(17) $$\begin{array}{c} \left[\begin{array}{cccc} B_{11} & B_{12} & B_{13} & -\lambda_{1}\\ B_{21} & B_{22} & B_{23} & -\lambda_{2} \end{array}\right]\left[\begin{array}{c} t_{C,x}^{N}\\ t_{C,y}^{N}\\ t_{C,z}^{N}\\ r_{L} \end{array}\right]=\left[\begin{array}{c} A_{11}t_{Cj,x}^{C_{i}}+A_{12}t_{Cj,y}^{C_{i}}+A_{13}t_{Cj,z}^{C_{i}}+t_{N,x}^{V}-C{\theta t}_{N,x}^{V}+S\theta t_{N,y}^{V}\\ A_{21}t_{Cj,x}^{C_{i}}+A_{22}t_{Cj,y}^{C_{i}}+A_{23}t_{Cj,z}^{C_{i}}+t_{N,x}^{V}-S\theta t_{N,x}^{V}-C\theta t_{N,y}^{V} \end{array}\right]. \end{array}$$

Consider Equation (17) over all frames. The final equation can be expressed as

(18) $$\begin{array}{c} \left[\begin{array}{cccc} B_{11} & B_{12} & B_{13} & -\lambda_{1}\\ B_{21} & B_{22} & B_{23} & -\lambda_{2}\\ \vdots & \vdots & \vdots & \vdots \\ B_{2N1} & B_{2N2} & B_{2N3} & -\lambda_{2N} \end{array}\right]\left[\begin{array}{c} t_{C,x}^{N}\\ t_{C,y}^{N}\\ t_{C,z}^{N}\\ r_{L} \end{array}\right]=\left[\begin{array}{c} A_{11}t_{Cj,x}^{C_{i}}+A_{12}t_{Cj,y}^{C_{i}}+A_{13}t_{Cj,z}^{C_{i}}+t_{N,x}^{V}-C{\theta t}_{N,x}^{V}+S\theta t_{N,y}^{V}\\ A_{21}t_{Cj,x}^{C_{i}}+A_{22}t_{Cj,y}^{C_{i}}+A_{23}t_{Cj,z}^{C_{i}}+t_{N,x}^{V}-S\theta t_{N,x}^{V}-C\theta t_{N,y}^{V}\\ \vdots \\ A_{2N1}t_{Cj,x}^{C_{i}}+A_{2N2}t_{Cj,y}^{C_{i}}+A_{2N3}t_{Cj,z}^{C_{i}}+t_{N,x}^{V}-S\theta t_{N,x}^{V}-C\theta t_{N,y}^{V} \end{array}\right], \end{array}$$

where the left matrix of the left term has 2N×4 dimensions; and the right term is a vector with 2N dimensions. *N* is a total number of frames, N=1, …,2N. From Equation (18), the head-eye translation and the actual radii of the left wheel, $\left(t_{C,x}^{N},t_{C,y}^{N},t_{C,z}^{N},r_{L}\right)$, are estimated using a linear least-squares method. The remaining parameters $r_{R}$ and *b* can be obtained with Equation (2). The wheel parameters, ($r_{L}$, $r_{R}$, *b*), which are obtained in this section, will be used to re-estimate the rotation between the robot’s head and neck, as previously described.

### 3.5. Linearly Iterative Estimation

Even though the previous approach [11] could estimate the odometric and head-eye parameters simultaneously, their method could not provide the completed six parameters. The translation of *z*-axis between head-eye coordinates, $t_{C,z}^{N}$ was not obtained by their method. The proposed method presents a fully mobile robot calibration of odometric and head-eye parameters, building on [14]. The rotation and translation in head-eye calibration including $t_{C,z}^{N}$ and the odometric parameters are simultaneously estimated precisely. Supposing that the values of all parameters are not obtained correctly before optimization, it leads all parameters to convergence with incorrect values or divergence. Therefore, this section explains our contribution that we apply iteration-based estimation to initially guess the values of all parameters correctly that leads all parameters to convergence with the correct values rapidly. The processes of Section 3.2, Section 3.3 and Section 3.4 are used to compute repeatedly until all parameters are converged. The head-eye parameters, $R_{N}^{C}$, are estimated from Section 3.2 prior to being used to calculate the intermediate wheel parameters, $\left(\alpha_{R},\alpha_{L}\right)$, of both wheels in Section 3.3. After that, they are used to compute the remaining parameters, as described in Section 3.4. These results are also used again to calculate the head-eye parameters following Section 3.2, as shown in Algorithm 1, which shows steps 3 to 5 compute repeatedly until convergence.

**Algorithm 1** Full algorithm simultaneous calibration for mobile robot with pan-tilt camera.
**Input:**
${\left\{T_{N_{i}}^{V_{i}}\right\}}_{i=1}^{i=N},\;{\left\{\phi_{R_{i}},\phi_{L_{i}}\right\}}_{i=1}^{i=N},\;I_{i=1}^{i=N}$

**Output:**
$R_{C}^{N},\;t_{C}^{N},\;r_{L},\;r_{R},\;b$
**for**i∈1,…,N−1**do** **Step 1:** Obtain $T_{C_{j}}^{C_{i}}$ between each frame using chessboard’s corners or natural features
**end for**
**Step 2:** Initial $r_{L},r_{R},b$ with manual measurements and obtain θ with Equation (3)**while** Convergence **do** **Step 3:** Compute $R_{C}^{N}$ with Equation (7) **Step 4:** Compute $\alpha_{R},\alpha_{L}$ with Equations (8)–(10) **Step 5:** Compute $t_{C}^{N},r_{L},r_{R},b$ with Equation (18)
**end while**
**Step 6:** Refine $R_{C}^{N},t_{C}^{N},r_{L},r_{R},b$ with Equation (19)

### 3.6. Non-Linear Optimization

Even though all parameters that are estimated in the previous section can lead to a good initial estimation, they are probably not the correct and accurate values. Therefore, a method of minimizing a function as Powell’s method [16] is applied to fine-tune all parameters as closely as the ground truth. The variables consisting of the Euler angles of the head-eye rotation, $R_{C}^{N}$, ($\gamma_{x},\gamma_{y},\gamma_{z}$), head-eye translation, ($t_{C,x}^{N},t_{C,y}^{N},t_{C,z}^{N}$), and wheel parameters, ($r_{L},r_{R}$, *b*) are refined using the following equation:

(19) $$\begin{array}{cc} \Theta^{★} & ={\arg\min}_{R_{C}^{N},t_{C}^{N},r_{L},r_{R},b}\;\;C\left(\Theta \right),\\ C(\Theta ) & =\sum_{i=1}^{N-1}\sum_{k=1}^{K}{\left[Q_{i,k}\;-\;Q_{i,k}^{\prime }\right]}^{2},\\ Q_{i,k}^{\prime } & =T_{N}^{C}T_{V_{i}}^{N_{i}}T_{V_{j}}^{V_{i}}T_{N_{j}}^{V_{j}}T_{C}^{N}{\left[X_{j}\;Y_{j}\;Z_{j}\;1\right]}^{T},\;j=i+1, \end{array}$$

where $Q_{i,k}^{\prime }$ is the predicted 3D features, which are used to transform any point *k* from frame *j*, j=i+1, to frame *i*. The 3D feature at frame *i*, represented with $Q_{i,k}$ and $T_{C}^{N}$, is constructed using head-eye rotational and translational parameters, ($\gamma_{x},\gamma_{y},\gamma_{z},t_{C,x}^{N},t_{C,y}^{N},t_{C,z}^{N}$). $T_{N}^{V}$ is calculated using DH parameters and pan-tilt data from the encoders; while $T_{V_{j}}^{V_{i}}$ is referred to $R_{z}\left(\theta \right)$, which is calculated using wheel encoder data and the estimated wheel parameters, $r_{L},r_{R},b$. *N* and *K* are the total number of images and features, respectively.

## 4. Experimental and Results

In our experiments, we used a mobile robot, which had two RGB cameras mounted on the pan-tilt motorized device, as shown in Figure 1. A chessboard and natural scenes were used as the calibration target for single camera and a pair of cameras (a stereo camera), respectively. The differential-drive mobile robot moved to any specified position and both the robot’s neck and pan-tilt axis also moved before capturing an image sequentially. The mobile robot moved and captured repeatedly to obtain a set of images, $I_{n=1}^{n=N}$. The data of the robot movements such as the rotational angles of both wheels ($\phi_{R_{n}}$, $\phi_{L_{n}}$) and of any image *n* ( *n* = 1, …, *N*) were obtained by their angular velocity ($\omega_{R_{n}},\omega_{L_{n}}$) and the movement period time, $\tau_{i}$, as shown in Equation (3). The angles of wheels at the starting position, *n* = 1, were determined with $\phi_{R_{1}}$ = $\phi_{L_{1}}$ = 0. The transformation, including rotation and translation between robot’s base and robot’s neck coordinate systems, $T_{N_{n=1,\ldots ,N}}^{V_{n=1,\ldots ,N}}$, were calculated with the rotational angles of the pan-tilt axis at the robot’s neck and Denavit–Hartenberg parameters (DH parameters).

In the case that used a chessboard as the calibration target, we captured a set of images using single camera with a resolution of 320×240 pixels. The chessboard contained 10 × 7 black and white square grids (56 corner points), and the size of any grid was 5.4×5.4 cm. We extracted the feature points of the chessboard’s corners manually using [17]. Since all feature points of the chessboard’s corners were obtained, the transformation $T_{C_{i}}^{W}$ between the camera at position *i* and the chessboard, which was determined to be the world coordinate, can be estimated with a plane-based transformation estimation [18]. Therefore, the transformation between any pair of camera positions *i* and *j*, $T_{C_{j}}^{C_{i}}$, was simply estimated with $T_{C_{j}}^{C_{i}}=T_{W}^{C_{i}}T_{C_{j}}^{W}$.

**Figure 4:**
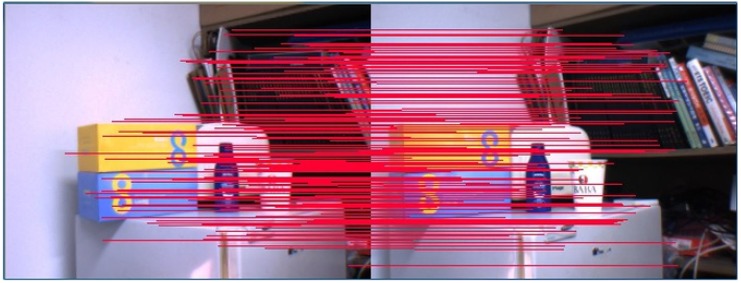
Natural features matching.

In the case of natural scenes, the natural features were observed from the real environment based on rectified images. The corresponding feature points of the stereo images were estimated with SURF [19]. The transformations between any pair of camera positions, $T_{C_{j}}^{C_{i}}$, were obtained by a closed-form solution of the least-squares problem of absolute orientation using orthonormal matrices [20]. The result of stereo matching is shown in Figure 4.

The transformations ($T_{C}^{N}$ and $T_{C}^{V}$), odometric parameters, and 3D back-projection error results of the proposed and Antonelli’s methods [11] were compared in Table 1. In the table of $T_{C}^{N}$, the head-eye rotation parameters ($\gamma_{x},\gamma_{y},\gamma_{z}$) were obtained by the ZYX-Euler angle corresponding to the rotation matrix $R_{C}^{N}$. The comparison of the head-eye transformation result indicated that the rotation and translation calibrated with the proposed method showed completed parameters estimation, while Antonelli’s method did not obtain the transformation between the camera and the robot neck due to the fact that their mobile robot’s neck could not move.

**Figure 5:**
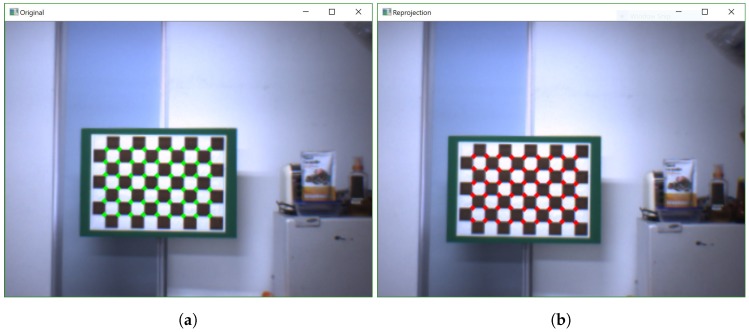
Reprojection results: (**a**) Reprojection of image *i*; (**b**) transformed reprojection of image *j*.

Furthermore, we also compared the results of the transformation between the robot’s base and the camera, $T_{C}^{V}$, by calculation of the transformation at the starting position, $T_{C_{1}}^{V_{1}}$, which was obtained by $T_{N_{1}}^{V_{1}}$ and $T_{C}^{N}$. The transformation matrix, $T_{C_{1}}^{V_{1}}$, of the proposed method was similar to Antonelli’s except the translation, $t_{C,z}^{V}$, that Antonelli’s method could not provide due to the constraining of the origin of the vehicle reference frame on the inertial x-y plane. The error was a 3D back-projection error, which was calculated with the average of Euclidean distance from all 3D feature points between any image and the transformed 3D feature points of other images, which was shown in mm units. Figure 5 also presents the reprojection result between frames of our method.

**Figure 6:**
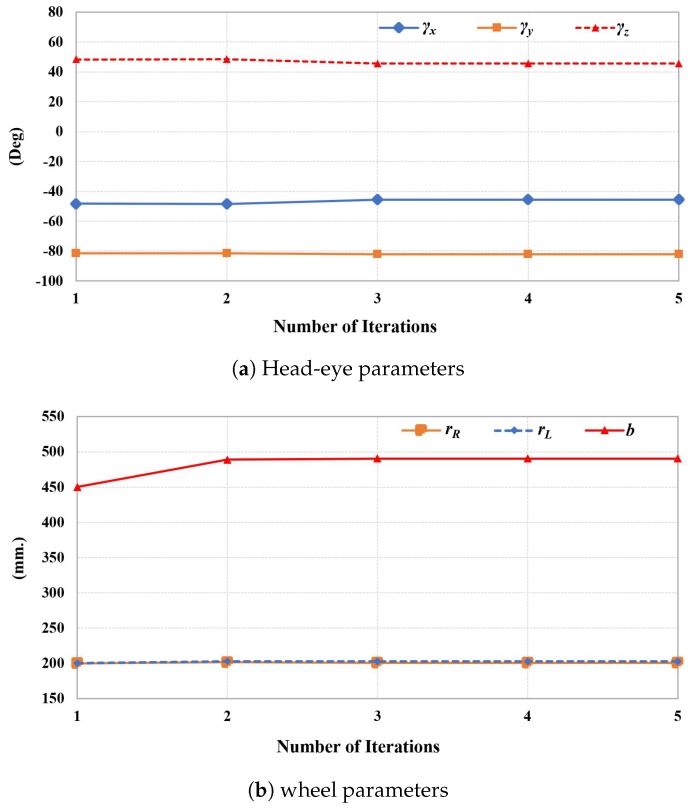
Rate of change related to number of iterative estimation.

**Figure 7:**
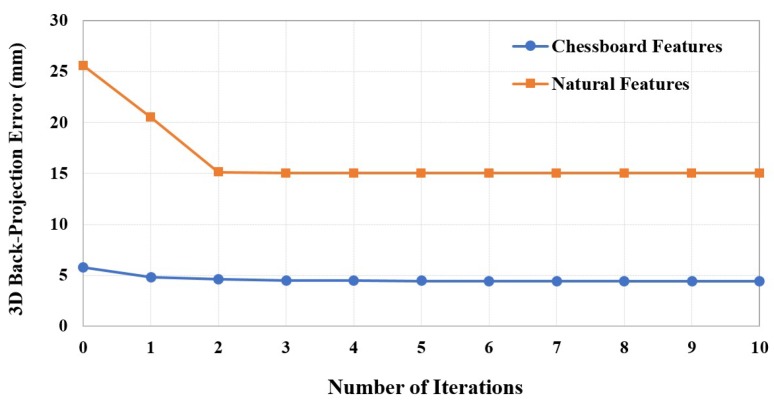
3D back-projection error after optimization related to number of iterations.

**Figure 8:**
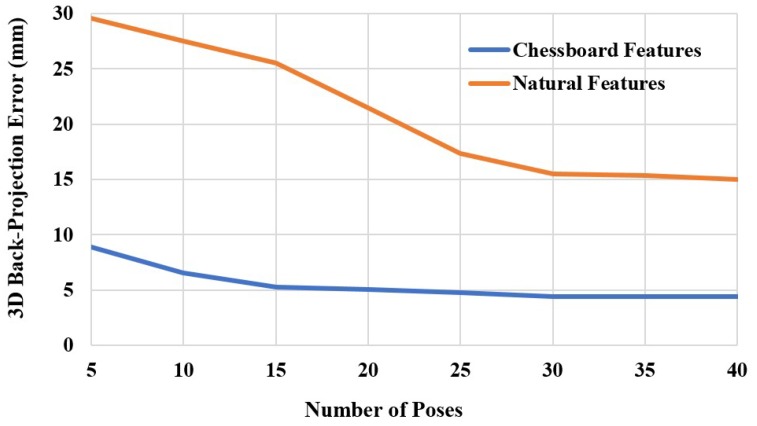
3D back-projection error after optimization related to number of poses.

Even though our method requires an iterative computation in Section 3.5, all parameters reach stability within just a few iterations, as shown in Figure 6. The calibration error after optimization using both chessboard and natural scenes with respect to the number of iterations is shown in Figure 7. However, the 3D back-projection error of the calibration using natural scenes also depends on accuracy of the stereo matching process. Although the back-projection error using natural features is significantly higher than using features from a chessboard, both cases required only a few optimized iterations before the error was steady, which demonstrated the back-projection error of 4.4239 mm, as represented in Table 1. The back-projection error before optimization (iteration = 0) and after optimization of both chessboard and natural scenes related to the number of images are represented in Figure 8. It shows the number of poses that affect to the calibration accuracy. However, the required number of input images using a chessboard as the calibration target is at least 30 input images, while using the natural features requires at least 35 input images for the steady results.

## 5. Conclusions

In this paper, we presented an approach for simultaneous calibration of head-eye and odometric parameters on the mobile robot equipped with a camera mounted on the motorized pan-tilt. Our proposed approach involves complete estimation of the wheel radii, wheelbase length, and the rotation and translation of the head-eye. Additionally, we obtain comprehensive results of the relative pose between the camera and the robot’s base, showing that our proposed method can compute the translation in *z*-axis while the previous studies could not. After the data from the visual features of either chessboard’s corners or natural scenes and odometry measurements were acquired, both head-eye and wheel parameters were simultaneously estimated by using iterative adjustment until all parameters converged—the experimental results showed a few iterations were necessary for the convergence. Furthermore, nonlinear optimization is used to minimize the cost function to more sufficiently and appropriate to perform on the mobile robot equipped with a pan-tilt camera precisely.

## Figures and Tables

**Table 1:** Results of the proposed method and Antonelli’s method.

Coordinate	Unit	Parameter	Proposed Method	Antonelli’s Method
$T_{C}^{N}$	Deg.	$\gamma_{x}$	−45.5715	-
		$\gamma_{y}$	−82.0160	-
		$\gamma_{z}$	45.4678	-
	mm.	$t_{C,x}^{N}$	31.5182	-
		$t_{C,y}^{N}$	−75.1629	-
		$t_{C,z}^{N}$	−90.0127	-
$T_{C}^{V}$	Deg.	$\gamma_{x}$	78.6669	−62.4582
		$\gamma_{y}$	−84.3044	−87.9764
		$\gamma_{z}$	11.0493	153.6164
	mm.	$t_{C,x}^{V}$	321.9080	221.4345
		$t_{C,y}^{V}$	−89.4305	−50.3979
		$t_{C,z}^{V}$	969.8691	unknown
wheel	mm.	$r_{L}$	202.4040	225.3074
		$r_{R}$	200.6111	227.3912
		*b*	490.4046	513.1268
Error	mm.		4.4239	7.9798
